# Multiparametric Profiling of Neutrophil Function via a High-Throughput Flow Cytometry-Based Assay

**DOI:** 10.3390/cells12050743

**Published:** 2023-02-25

**Authors:** Kyle D. Timmer, Daniel J. Floyd, Allison K. Scherer, Arianne J. Crossen, Johnny Atallah, Adam L. Viens, David B. Sykes, Michael K. Mansour

**Affiliations:** 1 Department of Medicine, Division of Infectious Diseases, Massachusetts General Hospital, Boston, MA 02114, USA; 2 Harvard Medical School, Boston, MA 20114, USA; 3 Center for Regenerative Medicine, Massachusetts General Hospital, Boston, MA 02114, USA; 4 Harvard Stem Cell Institute, Cambridge, MA 02114, USA; 5 Department of Stem Cell and Regenerative Biology, Harvard University, Cambridge, MA 02114, USA

**Keywords:** neutrophil, function, flow cytometry, high throughput assay, neutrophil augmentation

## Abstract

Neutrophils are a vital component of the innate immune system and play an essential function in the recognition and clearance of bacterial and fungal pathogens. There is great interest in understanding mechanisms of neutrophil dysfunction in the setting of disease and deciphering potential side effects of immunomodulatory drugs on neutrophil function. We developed a high throughput flow cytometry-based assay for detecting changes to four canonical neutrophil functions following biological or chemical triggers. Our assay detects neutrophil phagocytosis, reactive oxygen species (ROS) generation, ectodomain shedding, and secondary granule release in a single reaction mixture. By selecting fluorescent markers with minimal spectral overlap, we merge four detection assays into one microtiter plate-based assay. We demonstrate the response to the fungal pathogen, *Candida albicans* and validate the assay’s dynamic range using the inflammatory cytokines G-CSF, GM-CSF, TNFα, and IFNγ. All four cytokines increased ectodomain shedding and phagocytosis to a similar degree while GM-CSF and TNFα were more active in degranulation when compared to IFNγ and G-CSF. We further demonstrated the impact of small molecule inhibitors such as kinase inhibition downstream of Dectin-1, a critical lectin receptor responsible for fungal cell wall recognition. Bruton’s tyrosine kinase (Btk), Spleen tyrosine kinase (Syk), and Src kinase inhibition suppressed all four measured neutrophil functions but all functions were restored with lipopolysaccharide co-stimulation. This new assay allows for multiple comparisons of effector functions and permits identification of distinct subpopulations of neutrophils with a spectrum of activity. Our assay also offers the potential for studying the intended and off-target effects of immunomodulatory drugs on neutrophil responses.

## 1. Introduction

Neutrophils possess a repertoire of functions as the first line of defense in controlling invading pathogens. While the absolute neutrophil count is of obvious importance, the functional capacity of neutrophils to properly execute these functions is also critical for the prevention of disease [1,2,3]. Despite having normal or greater than normal neutrophil counts, studies suggest select patient cohorts face an increased risk of infection due to inherited or acquired defects in neutrophil function, including those with diabetes [4,5], cirrhosis [6,7], chronic granulomatous disease [8,9], and recipients of organ transplants [10]. Interrogating neutrophil functions can help to explain why neutrophils may be ineffective in controlling pathogens in these patients. For example, neutrophils from patients with diabetes have decreased reactive oxygen species (ROS) production, chemotaxis, phagocytosis, and neutrophil recruitment. Shedding light on these impairments may explain patients’ increased vulnerability to infection [11].

In addition to genetic and metabolic causes, many approved therapeutics can have unintended side effects leading to neutrophil dysfunction. Bruton’s tyrosine kinase (Btk) inhibitors, such as ibrutinib, a backbone in treating chronic lymphocytic leukemia [12], impairs neutrophil function [13]. Patients on treatment with ibrutinib have neutrophils with diminished phagocytosis, ROS generation, and cytokine production, which may explain the increased incidence of invasive aspergillosis in this patient population [13]. Developing an assay that rapidly and efficiently analyzes a diverse array of neutrophil functions could improve our understanding of neutrophil dysfunction in various disease settings and highlight potential off-target effects of medications on neutrophil function.

**Figure 1:**
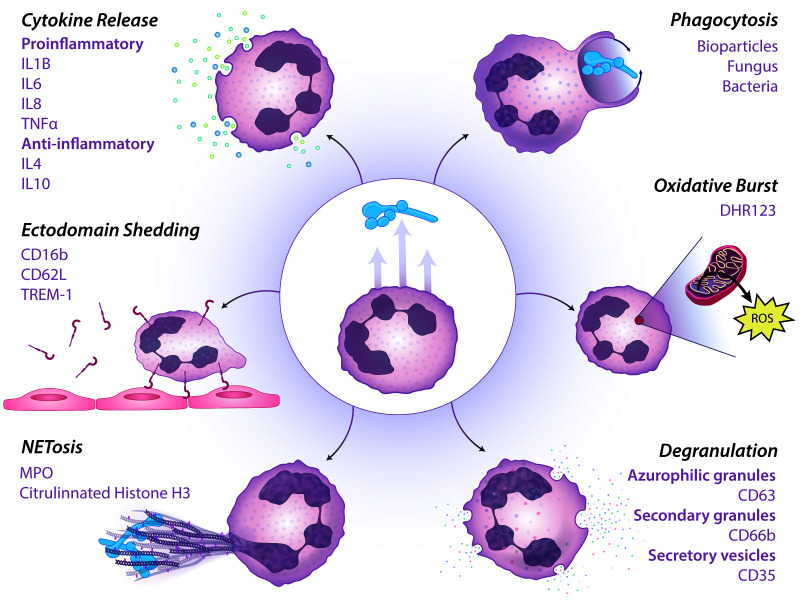
Measurable neutrophil functions by flow cytometry. Human neutrophils perform a variety of functions in response to pathogen sensing. Beneath each category or subcategory are surface proteins, soluble factors, dyes, or pathogenic insults that can be used to detect an array of neutrophil functions in a multiparametric flow cytometer-based assay. Illustration by Nicole Wolf, MS, ©2022. (nicolecwolf@gmail.com) Printed with permission.

Flow cytometry is commonly used for the concurrent measurement of multiple parameters on large populations of cells. Reactive dyes, fluorescent pathogens, analyte capture beads, and antibody labeling of cell surface proteins allow one to quantify various neutrophil functions (Figure 1) rapidly and can be accomplished within hours of blood collection [14]. Flow cytometry-based multifunctional assays have identified aberrant neutrophil activity in specific subpopulations of patients such as those with community-acquired pneumonia [14], HIV [15], or severe-injury-related trauma [16]. By quickly identifying patients with neutrophil dysfunction, interventions can be promptly employed to manage at-risk patients. To date, the breadth of measurements in such assays has been limited to two functions: phagocytosis and oxidative burst [14,15,16]. Specific protocols increase the study dimensions with parallel assays or downstream secondary analyses of supernatant to measure the release of soluble factors [17,18]. A high throughput assay that measures multiple functions remains an unmet need for granulocyte phenotyping. We present a microtiter plate assay for simultaneously measuring four neutrophil functions, including phagocytosis, ROS generation, ectodomain shedding, and degranulation.

The selected functions are cardinal features of neutrophil response. Ectodomain shedding, an often-overlooked neutrophil function, refers to the cleavage of specific motif-bearing surface proteins by selective proteases, most often of the a disintegrin and metalloproteases (ADAMs) family [19]. These changes to the neutrophil surface (ectodomain) influence various neutrophil activities such as cell-cell adhesion and rolling through the cleavage of L-selectin (CD62L) [20], opsonin recognition through the cleavage of the Fc receptor CD16b [21], paracrine signaling through the cleavage of tumor necrosis factor alpha (TNFα) and the TNFα receptors [22,23], and triggering neutrophil activation through the cleavage of the receptor TREM-1 [24,25]. CD62L is rapidly shed within minutes of stimulation [26] and is a classic and early indicator of neutrophil activation [27]. Additionally, CD62L, CD16b, TNFα, and the TNFα receptor are cleaved by the same metalloprotease, ADAM17 [28]. Thus, CD62L is an ideal marker for measuring ectodomain shedding because it acts as a metric for other surface changes and is an early sign of neutrophil activation.

Degranulation is another critical function in neutrophil pathogen response. Neutrophils possess granules that release preformed enzymes into the extracellular environment during degranulation [29]. These granules contain antimicrobials such as myeloperoxidase (MPO), lysozyme, lactoferrin, mixed metalloprotease 9, and neutrophil elastase [30]. Granule subsets are categorized based on their contents and are released in a regulated order: secretory vesicles, tertiary, secondary, and finally primary granules [31]. We chose to analyze secondary granule release, through expression of CD66b, since it requires greater activation than secretory vesicles and tertiary granules and because these granules contain multiple antimicrobial peptides such as lactoferrin, neutrophil gelatinase-associated lipocalin (NGAL), and prodefensin [30,32].

Here, we describe a new high-throughput assay that quantifies four neutrophil functions simultaneously from a single sample. By selecting compatible fluorescence read-outs, we minimize spectral overlap and demonstrate the potential to detect increases and decreases in neutrophil activity following treatment with cytokines, kinase inhibitors, and common immunomodulatory drugs. Our assay provides a convenient workflow for multiple rounds of priming or inhibition, pathogen co-culture, antibody labeling, and data acquisition within the same reaction well.

## 2. Materials and Methods

### 2.1. Reagents

Flow cytometry staining buffer (FACS buffer) was prepared with 2% heat-inactivated fetal bovine serum (FBS) (Life Technologies, Carlsbad, CA, USA) and 1 mM EDTA (Life Technologies) in phosphate-buffered saline (PBS) without calcium and magnesium (Corning, Corning, New York, NY, USA). Common immunosuppressive drugs were used to demonstrate the ability to test off-target therapeutic effects on neutrophil functions including cyclosporine A (10 μg/mL, Selleckchem, Houston, TX, USA) and mycophenolic acid (30 μM, Selleckchem). Cytokines used included G-CSF (100 ng/mL, Peprotech, Cranbury, NJ, USA), IFNγ (100 ng/mL, Peprotech), TNFα (10 ng/mL, Peprotech), and GM-CSF (10 ng/mL, Peprotech). Chemical inhibitors included diphenyleneiodonium for ROS inhibition (DPI, 10 μM, Selleckchem), TMI-005 for inhibition of CD62L shedding (2.5 μM, Cayman Chemical, Ann Arbor, MI, USA), PP1 for Src inhibition (10 μM, Cayman Chemical), R406 for Syk inhibition (20 μM, Selleckchem), and IBT for Btk inhibition (1 μM, Cayman Chemical). Lipopolysaccharide (LPS) from *E. coli* strain K12 was purchased from InvivoGen (San Diego, CA, USA). Complete RPMI (cRPMI) was prepared from RPMI (Corning), 10% FBS, 2 mM L-glutamine (Life Technologies), and 1% penicillin-streptomycin (Thermo Fisher Scientific, Waltham, MA, USA).

### 2.2. Preparation of Human Neutrophils

Healthy blood donors were consented under the Massachusetts General Hospital Institutional Review Board-approved protocol (2019P002840). Whole blood was collected in EDTA-coated vacutainers (Beckton Dickinson, Franklin Lakes, NJ, USA) and subsequently centrifuged at 1500xg for 15 min. Buffy coat was collected, and neutrophil isolation was performed using the negative selection EasySep Direct Human Neutrophil Isolation Kit, according to the manufacturer’s instructions (STEMCELL Technologies, Seattle, WA, USA). Wright-Giemsa staining was performed after the isolation process to confirm neutrophil purity from the isolation kit. Flow cytometry was also used to verify a high neutrophil purity from the isolation procedure (≥94% neutrophil purity). Cell concentration and viability were measured by staining the cells with a 1:10 dilution of acridine orange/propidium iodide followed by automatic cell counting using the LUNA fl Dual Fluorescence Cell Counter (Logos Biosystems, Annandale, VA, USA) (≥99% live). Neutrophils were resuspended in cRPMI at a concentration of 2 × 10^6^ cells/mL.

### 2.3. Preparation of C. albicans

Wildtype strain SC5314 *Candida albicans* was purchased from the American Type Culture Collection (American Type Culture Collection, Manassas, VA, USA). SC5314 constitutively expressing far-red fluorescent protein (*C. albicans* iRFP) was kindly donated by Robert Wheeler (University of Maine, Orono, ME) [33]. *C. albicans* was grown in YPD liquid media (yeast extract, peptone, dextrose) containing 1% yeast extract (Acros Organics, Fair Lawn, NJ, USA), 2% peptone (BD Biosciences, San Jose, CA, USA), and 2% dextrose (Sigma-Aldrich). *C. albicans* was cultured overnight at 30 °C on a rotating culture wheel (Thermo Fisher Scientific). The following day, *C. albicans* was removed from the wheel, washed twice with PBS and resuspended in PBS. *C. albicans* was counted using the LUNA automatic cell counter and kept on ice until the time of the assay.

### 2.4. Neutrophil-Candida Co-Incubation

Drugs, cytokines, or appropriate vehicles were prepared at a 2X concentration in cRPMI, with a maximum DMSO concentration of 0.1% *v*/*v*. In a 96-well V bottom polypropylene plate (Corning) the perimeter and outer wells were moated with 200 μL sterile PBS. Next, 50 μL of cRPMI or 50 μL of 2X drug or cytokine was mixed with 50 μL of neutrophil stock in a reaction well. The plate was sealed with breathable film (VWR, Radnor, PA) and placed in an incubator at 37 °C, 5% CO_2_ for 30 min to one hour. A 30-min cytokine incubation was chosen based on previous time courses. Neutrophils incubated with immunomodulatory drugs for 1 h, as this was the midpoint seen in similar published studies [34,35,36]. After the neutrophils were incubated, 20 μL of *C. albicans* at the desired multiplicity of infection (MOI) was added to the appropriate wells and mixed by pipetting. Immediately following, 30 μL of dihydrorhodamine 123 (DHR123) (5 μM, Thermo Fisher Scientific) was added to the wells and mixed well by pipetting. The plate was sealed and returned to the incubator for 30 min for coincubation with *C. albicans*. After the 30 min, the plate was placed on ice for 10 min in the dark and prepared for flow cytometry. During rescue studies, neutrophils were first treated with kinase inhibitors for 30 min [37] in an incubator. LPS (400 ng/mL) was then added to the well and the plate was returned to the incubator for an additional 45 min [37] prior to co-culturing neutrophils with *C. albicans* for 30 min.

### 2.5. Flow Cytometry

Cells were pelleted at 4 °C and stained in 50 μL of cold FACS buffer containing (BV605) anti-human CD62L antibody (1:200 dilution; clone DREG-56; BioLegend, San Diego, CA, USA) and (BV421) anti-CD66b (1:200 dilution; clone 6/40c; BioLegend). The cells were incubated for 30 min at 4 °C in the dark. Cells were rinsed with 150 μL cold FACS buffer, centrifuged at 4 °C and resuspended in 150 μL cold FACS buffer. The 96 well plate was left on ice until just prior to data acquisition on a BD FACSCelesta (BD Biosciences, San Jose, CA, USA) with a blue, violet, red (BVR) laser configuration with specific wavelengths at 488 nm, 405 nm, 640 nm respectively. Bandpass filters for the Celesta include 450/40, 525/50, 610/20, 660/20, 780/60 for the 405 nm laser; 530/30, 575/26, 610/20, 695/40 for the 488 nm laser; and 670/30, 730/45, 780/60 for the 640 nm laser. Before recording data, gates were prepared so that 7000 neutrophil events could be collected. Compensation was performed with single color controls and was calculated using BD FACSDiva Software (BD Biosciences). A compensation matrix demonstrating the spectral overlap values can be seen in Supplementary Table S1. FCS files were exported from BD FACSDiva Software in a 3.0 format. Analysis of FCS files was performed using FlowJo v.10 software (BD Biosciences). T-distributed stochastic neighbor embedding (tSNE) was performed in FlowJo on gated neutrophils and calculated with the four neutrophil function flow cytometer parameters. Heat map statistics for iRFP (phagocytosis), DHR123 (ROS), CD62L expression (shedding), and CD66b expression (degranulation) were then overlayed on tSNE plots. Fluorescence minus one (FMO) controls were performed to ensure minimal spectral overlap. For each FMO control, the multiparametric assay was run equivalently but the flow cytometry preparation excluded one fluorescent marker while retaining the others. Supplementary Table S2 provides further information on the fluorescence reagents and detection.

### 2.6. Statistical Analysis

All statistical analyses for normality and significance were performed on GraphPad Prism 9 (San Diego, CA) using ordinary one-way ANOVA. A *p*-value greater than 0.05 was considered nonsignificant (ns).

## 3. Results

**Figure 2:**
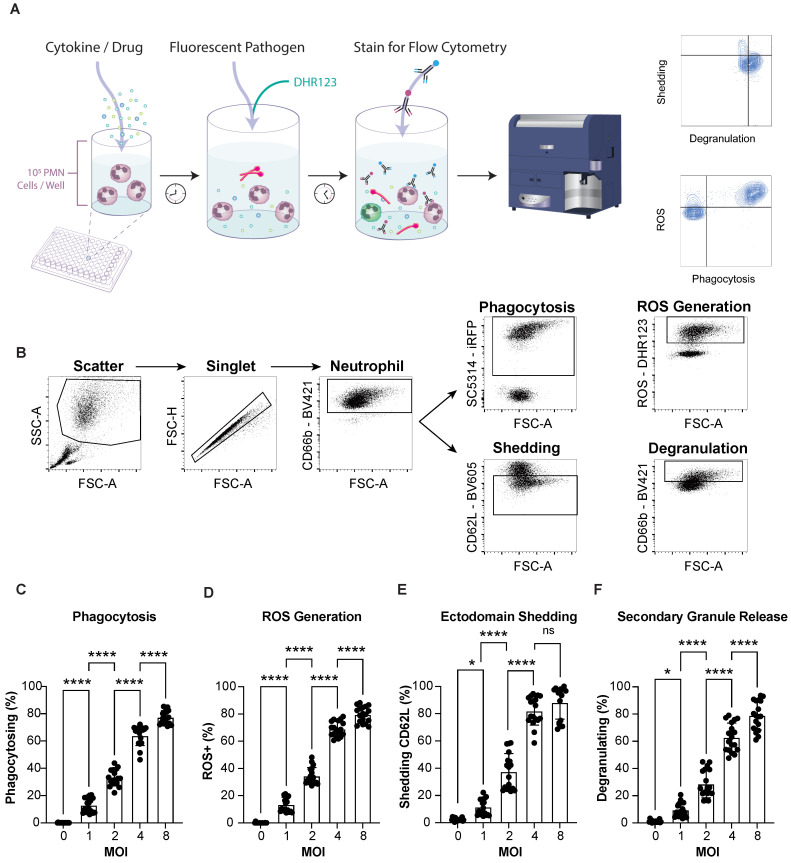
Multiple neutrophil functions can be measured using a multiparametric flow assay. (**A**) Schematic representation of assay workflow. Neutrophils are incubated with cytokines or drugs prior to the addition of ROS detection dye DHR123 and co-culture with iRFP expressing fungal pathogen *C. albicans*. After co-incubation, neutrophils are immunostained for the detection of secondary granule release and ectodomain shedding. Neutrophils were analyzed by flow cytometry for the frequency of functional behaviors. (**B**) Flow cytometry serial gating strategy for detection of neutrophil functions. Total events were first gated by scatter to target events with the proper size and granularity of neutrophils. Next, doublets were excluded, and finally, neutrophils were selected by gating for the expression of the neutrophil specific surface protein, CD66b (arrows designate sequential analysis). All function gates were set using fluorescence minus one controls, stained, unstimulated samples. (**C**–**F**) Percentage of total neutrophils exhibiting phagocytosis (**C**), ROS generation (**D**), ectodomain shedding (**E**), or secondary granule release (**F**) in response to increasing ratios of *C. albicans*. Four different multiplicities of infection (MOI), 1, 2, 4, 8 were tested. Data are represented as mean ± SD; *n* = 3 per group in each experiment per donor; experiments were repeated six times with six different donors. Data were analyzed by one-way ANOVA with a Tukey posttest. * *p* < 0.03, **** *p* < 0.0001. Illustration by Nicole Wolf, MS, ©2022. (nicolecwolf@gmail.com) Printed with permission.

Neutrophils employ a variety of effector functions to combat pathogens including phagocytosis, oxidative burst, degranulation, cytokine release, ectodomain shedding, and NETosis as illustrated in Figure 1. Here, we demonstrate a simple multiparametric assay to examine four neutrophil functions to *Candida albicans* challenge. Our assay is performed in a 96 well plate format and requires 1 × 10^5^ cells per well. Neutrophils isolated from healthy donors or patients can be pre-incubated with test biologics or small molecules to assess their influence on neutrophil functions. Following pre-incubation, fluorescent *C. albicans* are introduced along with DHR123 as an indicator for ROS production. Following co-incubation with *C. albicans*, neutrophils are immunostained for CD62L and CD66b. CD62L allows for the detection of ectodomain changes and elevation of CD66b serves as a metric for secondary granule release (Figure 2A).

A stepwise flow cytometric gating strategy identified neutrophils. Total events were gated by forward and side scatter properties to target live cells with the correct size and granularity. This gate removed most free-floating *C. albicans* and non-granulocytes (Figure 2B). It is important to note that this initial gate was generous because activated neutrophils engaging in the various functions tend to increase in size (FSC) and granularity (SSC). Single cells were identified, and neutrophils were selected by their expression of the neutrophil-specific surface protein CD66b (Figure 2B). Gates were set by comparing fluorescence minus one control to unstained as well as unstimulated stained samples. Phagocytosing neutrophils were gated as iRFP(+) events, ROS producing neutrophils were gated as DHR123(hi) events, shedding neutrophils were gated as CD62L(-) events, and degranulating neutrophils were gated as CD66b(hi) events. Neutrophil functions were analyzed simultaneously (Figure 2A) or individually (Figure 2B).

Increasing the ratio of *C. albicans* to neutrophils in the assay format increased the frequency of cells responding in terms of ectodomain shedding, phagocytosis, degranulation, and ROS production (Figure 2C–F). The multiplicity of infection (MOI) of 1, 2, 4, and 8 were statistically different in the frequencies of neutrophils engaging in phagocytosis, ROS generation, and secondary granule release (Figure 2C,D,F), while CD62L shedding plateaued between MOI or 4 and MOI of 8 (Figure 2E). Vastly different levels of neutrophil activity were achieved simply by increasing the ratio of *C. albicans* to neutrophils. Using an MOI of 8 triggered robust neutrophil responses resulting in 77% phagocytosis and 79% secondary granule release compared to 33% and 29%, respectively, at an MOI of 2 (Figure 2C,F).

**Figure 3:**
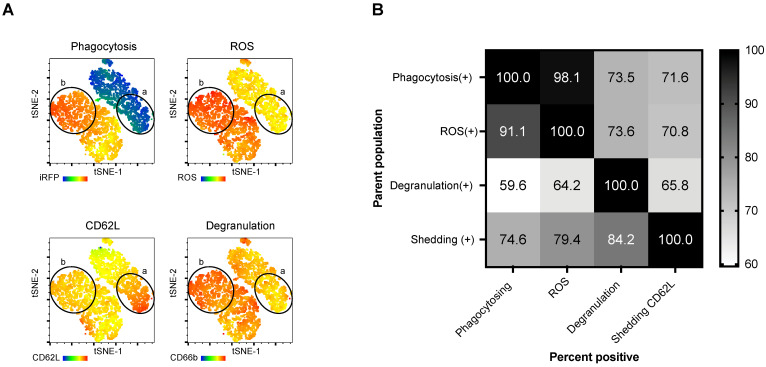
The multiparametric assay allows for the detection of neutrophils performing combinations of simultaneous functions. (**A**) tSNE projection of neutrophils incubated for 30 min with MOI of 4 *C. albicans*. Heatmap statistics of each flow cytometry parameter were individually overlayed to visualize low functioning (a) and multifunctioning (b) neutrophil subpopulations. (**B**) Neutrophils were co-cultured with *C. albicans* (MOI of 2) for 30 min. Neutrophil populations can be compared for activity in multiple functions. Above, neutrophils are gated on a single-parent function. Subsequently, we show the frequency of neutrophils from the parent function concurrently exhibiting a second function. Each row represents the positive population of neutrophils for a given parent function. Each column represents the mean percentage of neutrophils from the parent gate undergoing an additional function. *n* = 3 per experiment per donor; experiments were repeated four times with four different donors.

Many neutrophil functional studies have examined phagocytosis and ROS production [14,15], while our assay simultaneously measures four canonical neutrophil functions. The multiparametric nature of the assay reveals that not all neutrophils perform the exact coordination of functional responses (Figure 3A). For example, shedding and degranulation may occur regardless of whether a neutrophil is phagocytosing and generating ROS. Further, some neutrophils may be performing all four simultaneous functions (population b) while others have not begun any of the measured responses (population a). We additionally, determined the overlap of neutrophils that could participate in more than one function. Figure 3B summarizes the proportion of individual neutrophils engaging in multiple simultaneous functions after co-culture with *C. albicans*. This presentation of the data reveals relationships between neutrophil functions. For example, there was great overlap between phagocytosing and ROS-generating neutrophils. Most phagocytosing neutrophils also generated ROS; however, only about 60% of degranulating neutrophils were simultaneously phagocytosing *C. albicans*. Furthermore, our assay allows one to measure the frequency of neutrophils participating in four concurrent functions. At an MOI of 1, on average, 40% of phagocytosing neutrophils participate in all four functions simultaneously compared to 82% at MOI of 4.

**Figure 4:**
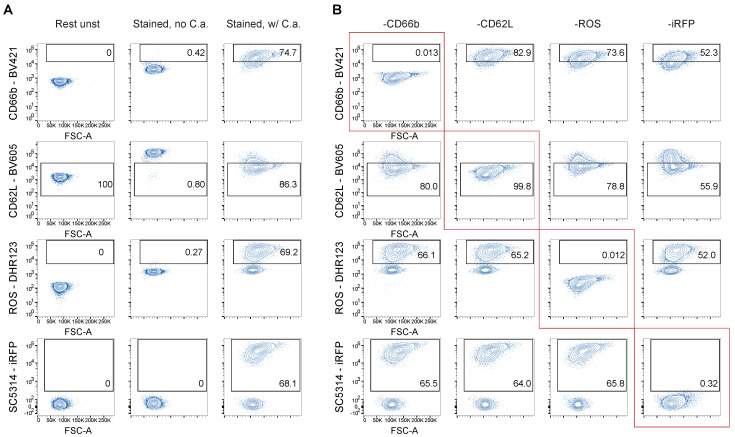
Fluorescence minus one (FMO) controls prove no confounding spectral overlap. (**A**,**B**) Resting neutrophils (no C.a) and *C. albicans* challenged neutrophils (w/C.a.) were stained (**A**) and compared to FMO controls (**B**), for which one fluorescent marker was removed at a time while the others were held constant. Red box denotes the FMO and the corresponding channel.

Each flow cytometer is equipped with multiple lasers and filters that allow different fluorescent panel possibilities. The cytometer configuration is carefully paired with fluorescent markers to minimize spectral overlap and the possibility of false positives. To ensure our multiparametric assay accurately detected individual functions, gates were set using fluorescence minus one (FMO) controls. In the degranulation (BV421), ROS (DHR123), and phagocytosis (iRFP) FMO controls, there were fewer than 0.4% positive events for each function (Figure 4A,B). In the ectodomain shedding (BV605) FMO control, 99.8% of neutrophil events were negative for CD62L expression (Figure 4A,B). Our assay reliably measures individual functions, and results are not confounded by spectral overlap.

**Figure 5:**
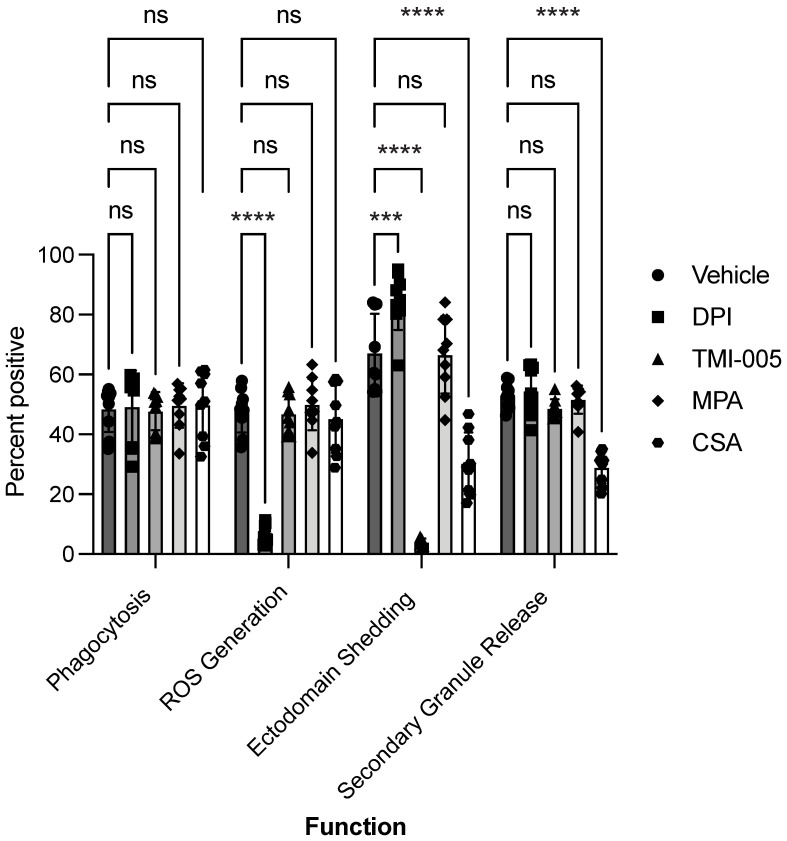
The multiparametric assay screens for modulation of neutrophil function by therapeutic and inhibitory drugs. Neutrophils were pre-treated with functional inhibitors TMI-005 or diphenyleneiodonium (DPI) or the immunosuppressive drugs cyclosporine A (CSA) or mycophenolate (MPA) for one hour. Neutrophils were then co-cultured with *C. albicans* (MOI of 3) for 30 min. The frequency of total neutrophils performing phagocytosis, ROS generation, ectodomain shedding, or secondary degranulation was measured. Data are represented as mean ± SD; *n* = 3 per group in each experiment per donor; experiments were repeated three times with three different donors. Data were analyzed by one-way ANOVA with a Tukey posttest. *** *p* < 0.0002, **** *p* < 0.0001, ns = *p* > 0.05.

An essential application of the multiparametric assay is to screen potential bioactive small molecule drugs for neutrophil-modulating effects. We selected a panel of compounds known to influence immune function. These chemicals are inhibitors of ROS production (DPI) [38], and ectodomain shedding (TMI-005) [20], as well as two clinically relevant compounds used for immune suppression in transplant patients, mycophenolate (MPA) [39] and cyclosporine A (CSA) [40]. Treatment with compounds for one hour before *C. albicans* co-culture did not significantly affect neutrophil phagocytic ability. DPI was the only chemical to profoundly affect ROS generation, decreasing the number of ROS-positive cells by 87% (Figure 5). Ectodomain shedding was influenced by many of the small molecule compounds tested. The ADAM17 inhibitor TMI-005, nearly abolished ectodomain shedding. Treatment with MPA did not cause significant impairments to neutrophil functions. CSA however did cause a striking 55% reduction in ectodomain shedding and 45% reduction in secondary granule release. The small molecules tested resulted in unique changes to specific neutrophil behaviors and highlight the value of determining the precise functional area of neutrophil impairment.

**Figure 6:**
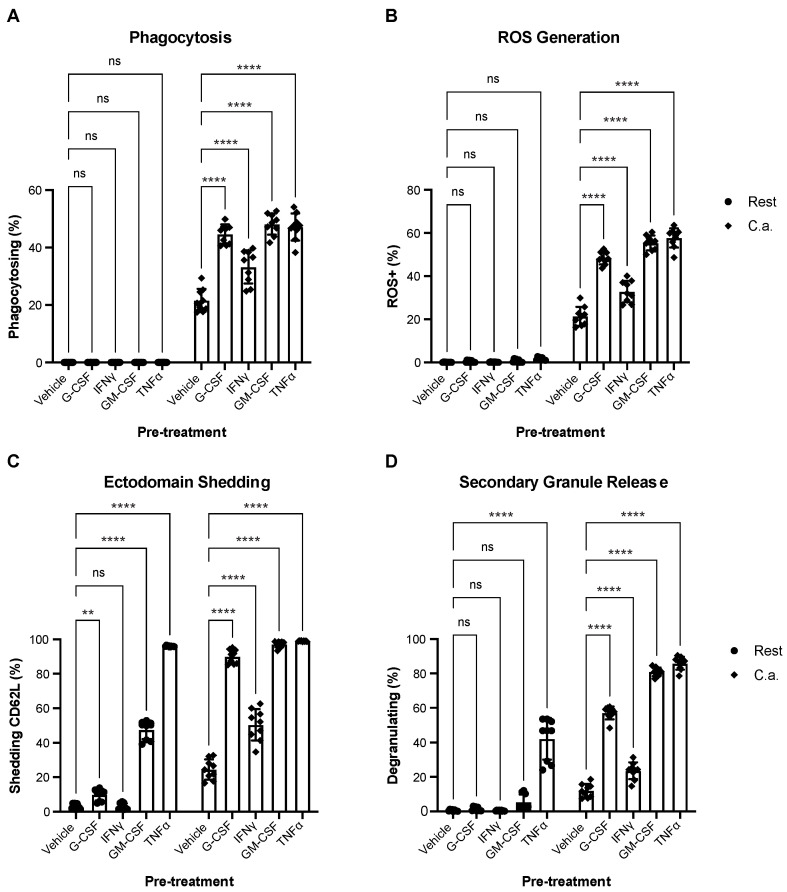
The multiparametric assay can detect functional augmentation. Neutrophils were pre-treated with pro-inflammatory cytokines G-CSF, IFNγ, GM-CSF, TNFα or vehicle control for 30 min to prime the neutrophils for enhanced functional output before coincubation with *C. albicans* (MOI of 2). The frequency of total neutrophils performing phagocytosis (**A**), ROS generation (**B**), ectodomain shedding (**C**), or secondary degranulation (**D**) was measured at rest (Rest) and after co-culture (C.a.). Data are represented as mean ± SD; *n* = 3 per group in each experiment per donor; experiments were repeated three times with three different donors. Data were analyzed by one-way ANOVA with a Tukey posttest. ** *p* < 0.0021, **** *p* < 0.0001.

To demonstrate the ability to measure neutrophil augmentation, we pre-stimulated neutrophils with cytokines, including granulocyte colony-stimulating factor (G-CSF), interferon-gamma (IFNγ), granulocyte-macrophage colony-stimulating factor (GM-CSF), and tumor necrosis factor-alpha (TNFα). These four inflammatory cytokines significantly increased the frequency of neutrophils engaging in phagocytosis, ROS generation, ectodomain shedding, and secondary granule release relative to the vehicle (Figure 6A–D). The response was nonuniform and cytokine-specific; IFNγ elicited the least improvement in neutrophil function. While IFNγ increased secondary granule release, the response was less than that of G-CSF, GM-CSF, or TNFα stimulation. Additionally, some of these cytokines began to trigger neutrophil responses in the absence of *C. albicans* co-culture. TNFα and GM-CSF produced significantly more ectodomain shedding at rest (Figure 6C). Interestingly, GM-CSF and TNFα encouraged degranulation in approximately 80% of neutrophils cultured with *C. albicans* (Figure 6D), nearly a sevenfold increase over the vehicle control. TNFα stimulation was unique in that it induced significant secondary granule release in the absence of a pathogen, a result seen in other studies [41].

**Figure 7:**
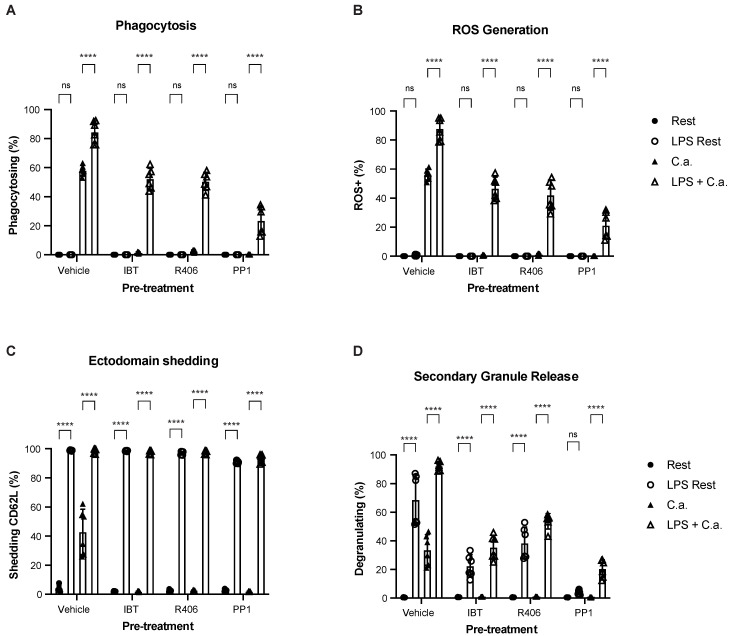
Multiparametric assay provides a simple format for measuring recovery of function in chemically inhibited neutrophils. Neutrophils were pre-treated with kinase inhibitors ibrutinib (IBT), R406, PP1 or DMSO vehicle control (Vehicle) for 30 min in the reaction well. Lipopolysaccharide (LPS, LPS + C.a.) or the LPS vehicle (Rest, C.a.) was then spiked into the reaction well and neutrophils incubated for an additional 45 min. Finally, neutrophils were co-cultured with *C. albicans* (MOI of 4) for 30 min (C.a., LPS + C.a.). The frequency of total neutrophils performing phagocytosis (**A**), ROS generation (**B**), ectodomain shedding (**C**), or secondary degranulation (**D**) was measured at rest (Rest) and after co-culture (C.a.). Data are represented as mean ± SD; *n* = 3 per group in each experiment per donor; experiments were repeated two times with two different donors. Data were analyzed by one-way ANOVA with a Tukey posttest. **** *p* < 0.0001.

We also sought to determine how the assay might be used to study the recovery of functional responses in dysfunctional or attenuated neutrophils. Inhibition of downstream kinases (e.g., Btk, Syk, Src) within the Dectin-1 signal transduction pathway can render neutrophils unresponsive to fungal pathogens such as *C. albicans* or *Aspergillus fumigatus* [42,43]. Syk and Btk inhibition can completely block neutrophil functions, including swarming, phagocytosis, oxidative burst, and cytokine production, even when challenged with *C. albicans* or *A. fumigatus* [37,44,45]. In the context of our assay, the inhibition of Btk, Syk, and Src, prevented phagocytosis, ROS production, ectodomain shedding, and secondary granule release (Figure 7A–D, respectively).

Neutrophil inhibition by ibrutinib (IBT) or R406 can be overcome by alternate pathway stimulation (e.g., TLR stimulation using LPS) before challenging with *C. albicans* [37]. LPS stimulation alone did not trigger ROS production in pre-treatment conditions when *C. albicans* was not present (Figure 7B). However, when inhibited neutrophils were stimulated with LPS and challenged with *C. albicans*, phagocytosis, and ROS production were significantly restored (Figure 7A,B). All LPS-treated neutrophil conditions had near unanimous shedding of CD62L (Figure 7C), and secondary granule release was improved with LPS stimulation (Figure 7D). These results suggest that the multiparametric assay can measure inhibition, augmentation, and subsequent pathogen challenge in the presence of crucial kinase inhibitors.

## 4. Discussion

While the absolute number of circulating neutrophils is critical for pathogen control, the neutrophil function is another crucial factor that is less easily quantified. Microbial killing assays provide information on the overall ability of neutrophils to recognize and eliminate pathogens, though they do not explain which specific functions contribute to pathogen detection and containment. In studying how specific disease states and small molecules may influence pathogen killing, we wished to quantify multiple neutrophil functions simultaneously. Here, we have demonstrated a simple high-throughput multiparametric flow cytometer-based assay that permits studying four canonical neutrophil functions: phagocytosis, ROS production, ectodomain shedding, and secondary granule release. Furthermore, we demonstrated our assay’s clinical relevance and sensitivity in detecting improvements and diminution of neutrophil activities by pre-stimulating neutrophils with biological and small molecule agents.

Our study does have limitations. There is growing evidence of neutrophil heterogeneity in peripheral blood [46], suggesting there may be populations with distinct functional potentials [47]. Our multiparametric assay did not include markers, such as CD10, to identify left shift neutrophils, an immature subset known to behave differently than mature circulating neutrophils [48,49]. Our study focused on healthy individuals who tend to contain scarce populations of immature neutrophils in circulation [48]. While it is unlikely that a left-shift neutrophil population significantly influenced the results demonstrated here, future work with the multiparametric assay should incorporate heterogeneity markers to fractionate results into additional unique subsets.

Our assay is not exhaustive of all neutrophil functions, and future iterations could be expanded to define additional neutrophil responses (Figure 1). The most versatile flow cytometers can be equipped with up to nine lasers and corresponding detectors, allowing for the theoretical recognition of ~40 different parameters. Our assay did not include a measurement of NETosis, a well-defined mechanism for pathogen control. During NETosis, neutrophils ensnare the microbe in a conglomerate of nuclear material laced with high concentrations of antimicrobial proteins [50,51]. NETosis can be quantified by flow cytometry using cell impermeable nucleic acid dyes such as SYTOX and fluorochrome-conjugated antibodies targeting MPO and/or citrullinated histone 3 [52,53] (Figure 1). In addition to pathogen elimination, measuring relative neutrophil extracellular trap formation is also essential for understanding potential tissue damage in the host. Excessive NET formation is implicated in ongoing endothelial tissue damage [54] and can contribute to microthromboses [55,56].

Further adaptations to the panel could also include readouts of other granule types. For example, primary (also known as azurophilic) granule release results in the extracellular expulsion of toxic proteins such as proteinase 3, neutrophil elastase, and MPO [30]. Primary granule release can be measured using fluorochrome conjugated antibodies against CD63 which is not traditionally expressed on the neutrophil surface but is deposited on the cellular membrane upon primary granule fusion (Figure 1) [57,58,59]. In developing future multiparametric assays to measure neutrophil function, primary granules are of key interest in bacterial pathogenesis. Some pathogens inhibit primary granule release as an immune evasion strategy [60]. Furthermore, the recognition of excessive primary degranulation of neutrophil elastase has been implicated in host tissue damage [61]. Alternatively, secretory granule release can be measured by similarly detecting increases in the surface level expression of CD11b or CD35, though these responses can be non-specifically provoked making them markers of general neutrophil activation [32,62].

The discarded reaction media from the neutrophil-pathogen co-culture contains informative metabolites and biomolecules that could shed light on specific neutrophil responses including the measurement of cytokines involved in cell-cell communication. Advancements in multiplex cytokine panels could allow for multiple soluble analyte measurements from microtiter volumes, making it compatible with the miniaturized design of our assay. Shed compounds such as CD62L, TREM-1 or the release of factors like G-CSF, or other pro and anti-inflammatory cytokines could be measured by bead capture and quantified by flow cytometry [63,64].

In our assay, we used live *C. albicans* marked by the constitutive expression of a fluorescent protein. Enforced expression of fluorescent proteins by pathogens are useful tools however, genetic manipulation may introduce additional changes from the parental strain [65]. In our FMO experiment, lower rates of all functions were observed, when the nonfluorescent parental strain of *C. albicans* was incubated with neutrophils. This result was obtained on multiple separate occasions and highlights a limitation in utilizing fluorescent protein expressing pathogen. Live fluorescent pathogen culture is not feasible in all lab settings and instead, uncolored pathogens may be conveniently FITC stained [66] or surface labeled using succinimidyl ester-based reactions [37]. Live pathogens may also not be feasible, or approved, for use in cytometer or within every flow cytometry core facility. If so, the quantification of phagocytosis can be adapted to measure the uptake of alternative targets such as fixed or heat-killed samples. Additionally, inert fluorescent bioparticles or fluorescent beads coated with microbial antigens could also substitute the use of live pathogens [17,67,68].

The multiparametric assay accurately measures augmented neutrophil functions. Cytokine therapies have been explored for multiple patient diagnoses, such as cirrhosis [10,69] and cases of neutrophil dysfunction [70]. Cytokine screens in our multiparametric assay could identify the most effective treatments for enhancing or attenuating neutrophil activity for specific diagnoses and therefore increase patient outcomes. Our assay design may also be applied to studies investigating the role of endogenous cytokine levels in tissue-specific microenvironments in the setting of diseases such as cystic fibrosis [71] or cancer [71,72].

Newly evolving biologic and small molecules are constantly being developed for the treatment of a variety of autoimmune disorders and malignancies. Small molecule kinase inhibitors have revolutionized such treatments; however, they can have off-target effects and can leave the patient vulnerable to invasive fungal disease [73]. We showed that three kinase inhibitors (IBT, R406, and PP1) eliminate neutrophil responses to *C. albicans* but that function can be restored with cytokines and growth factors. In the development of novel drugs, our assay could be used to detect negative consequences of kinase inhibiting therapeutics on neutrophil function and potential ways to overcome function inhibition.

In addition to kinase inhibitors, we tested two FDA-approved drugs, CSA and MPA, that are frequently used to suppress T and B cells [74,75] in transplant patients. The calcineurin inhibitor, CSA, suppressed innate immune cell function including neutrophil ectodomain shedding and secondary granule release. Other studies have also observed inhibitory effects of CSA on neutrophil function in vitro and in vivo, including reductions in primary granule release, neutrophil migration, and ROS production [34,35]. Our assay did not detect a decrease in ROS production though it appears that CSA diminishes ROS production in response to some but not all neutrophil-stimulating molecules [34]. This distinction may also reflect the type of ROS detected, specifically those from external release versus internal signaling [76,77].

As our assay detects functions simultaneously, one can observe changes to coupled mechanisms. Figure 3 shows a substantial overlap between individual cells capable of phagocytosis and ROS production. The strong association between phagocytosis and ROS-producing cells can be explained by the fact that NOX2 is activated upon phagosome formation [78,79]. When neutrophils were treated with the ROS inhibitor DPI, phagocytosis was relatively unchanged, but ROS was nearly abolished, highlighting the ability to investigate uncoupled functional processes.

Our assay yields reproducible results and possesses numerous applications for studying functional deficits. Larger donor enrollment could establish a healthy neutrophil function range from which aberrant neutrophil activity could be identified for clinical applications. We showed that our multiparametric assay could detect function improvement upon pre-incubation with inflammatory cytokines. Our assay may therefore be compatible with studies looking to augment neutrophil responses in patients experiencing neutrophil dysfunction or recurrent opportunistic infections [10,70].

In conclusion, our multiparametric assay demonstrates the utility of simultaneous measurement of several neutrophil functions that can be used for patient and population cohort immune profiling, assessment of biological or small molecule impact on granulocyte function as well as determination of novel approaches to augmentation of patients with dysfunctional neutrophils for improved health outcomes. The potential addition of metrics such as NETosis and cytokine release can render these assays more informative.

## Data Availability

Access to raw data files is available upon request.

## Supplementary Materials

The following supporting information can be downloaded at: https://www.mdpi.com/article/10.3390/cells12050743/s1, Supplementary Table S1. Compensation matrix with spectral overlap values calculated using BD FACSDiva software; Supplementary Table S2. Fluorescence reagent descriptions.
