# Supplementary material for: Multiparametric Profiling of Neutrophil Function via a High-Throughput Flow Cytometry-Based Assay
Source: Cells. 2023 Feb 25;12(5):743. doi: 10.3390/cells12050743 (PMC10000770; doi:10.3390/cells12050743)
Supplement: Supplementary file 1 [file cells-12-00743-s001.zip › Supplementary Table 1.pdf]

		-% Fluorochrome			
		**iRFP**	**BV421**	**BV605**	**DHR123**
**Fluorochrome**	**iRFP**	100	0.02	0.01	0.03
	**BV421**	2.75	100	0.87	0.78
	**BV605**	10.45	0.53	100	8.88
	**DHR123**	1.06	0.00	0.00	100
