# Supplementary material for: Multiparametric Profiling of Neutrophil Function via a High-Throughput Flow Cytometry-Based Assay
Source: Cells. 2023 Feb 25;12(5):743. doi: 10.3390/cells12050743 (PMC10000770; doi:10.3390/cells12050743)
Supplement: Supplementary file 1 [file cells-12-00743-s001.zip › Supplementary Table 2.pdf]

**Characteristic(s) being measured**	**Analyte**	**Analyte detector**	**Reporter**	**Laser (Bandpass filter)**
Cell type (Neutrophil)	CD66b	Anti-CD66b	BV421	405 (450/40)
Phagocytosis	Intracellular *C. albicans*	*C. albicans* constitutively expressing Near-infrared fluorescent protein (iRFP)	iRFP	640 (670/30)
Oxidative burst	Reactive oxygen species	Dihydrorhodamine 123 (DHR123)	Rhodamine 123	488 (530/30)
Ectodomain shedding	CD62L	Anti-CD62L	BV605	405 (610/20)
Degranulation	CD66b	Anti-CD66b	BV421	405 (450/40)
